# The immunologically high-risk kidney recipient in the early post-COVID-19 period. To do or not to do? A case report

**DOI:** 10.3389/fmed.2023.1147835

**Published:** 2023-03-23

**Authors:** Oana Antal, Alina Daciana Elec, Adriana Muntean, Tudor Moisoiu, Razvan Marian Melinte, Florin Ioan Elec

**Affiliations:** ^1^Department of Anaesthesia and Intensive Care, Iuliu Haţieganu University of Medicine and Pharmacy, Cluj-Napoca, Romania; ^2^Clinical Institute of Urology and Renal Transplantation, Cluj-Napoca, Romania; ^3^Department of Urology, Iuliu Haţieganu University of Medicine and Pharmacy, Cluj-Napoca, Romania; ^4^Department of Anatomy, Dimitrie Cantemir University, Târgu-Mureş, Romania; ^5^Department of Orthopedics and Trauma Surgery, Humanitas Hospital, Cluj-Napoca, Romania

**Keywords:** kidney transplant, COVID-19, hyper-immunization, recent COVID-19, SARS-CoV-2

## Abstract

Kidney transplantation is nowadays the treatment of choice for end-stage kidney disease (ESKD), and it is the most performed organ transplantation. During the COVID-19 pandemic, kidney-transplant recipients appeared to be at higher risk of morbidity and mortality due to severe forms of illness. The result was a decrease in the number of solid organs transplants worldwide, with patients' reduced chance of receiving transplants. The best timing for surgery after COVID-19 infection is still controversial since most of the available data come from study periods with zero or low prevalence of vaccination and COVID-19 variants with high mortality rates. The American Society of Anesthesiologists (ASA) and the Anesthesia Patient Safety Foundation (APSF) Joint Statement on Elective Surgery/Procedures and Anesthesia for Patients after COVID-19 Infection states that elective surgery should be delayed for 7 weeks after a SARS-CoV-2 infection in unvaccinated patients while making no clear statement for vaccinated ones, or those which have already been infected with the virus. Kidney transplant, as opposed to tissue transplant, is not an elective surgery, so the question raised is whether to do it or not. We present the case of a hyper-immunized 47-year-old male patient with end-stage chronic kidney disease who received a second kidney transplant, despite having a mild SARS-COV 2 infection just 2 weeks before his transplantation surgery.

## Introduction

COVID-19 pandemic extensively impacted the management of end-stage CKD patients and immunocompromised patients, as they have been proven to be more susceptible to more severe forms of pneumonia and complications due to frequent hospital visits and low immune capacity (1). Questions have been raised regarding patient safety and prognosis for a kidney transplant in patients recovering from COVID-19. There have been several publications on this matter (2, 3), but the general impression is that we need more data to draw a higher evidence conclusion. These patients underwent anesthesia and transplantation after at least 4 weeks following the SARS-CoV-2 infection.

We present the case of a hyper-immunized 47-year-old male patient with end-stage chronic kidney disease that has received a second kidney transplant, despite having had a recent mild SARS-CoV-2 infection.

## Case description

A 47-year-old male patient was admitted to our hospital on the 15th of September 2022 for a second **cadaver donor kidney transplant** evaluation. He presented with 15 years of dialysis-dependent end-stage chronic kidney disease following a hepatitis C cryoglobulinemic nephropathy and a chronic rejection of a previous living donor kidney transplant he had received in 2002.

He was considered hyper-immunized due to his previous graft and calculated Panel Reactive Antibodies of 86% (cPRA) (4). In addition, he had a history of **hepatitis C virus (VHC)**, hepatitis **B virus (VHB)**, and pancytopenia in a hypersplenism context (two bone marrow biopsies with no pathological findings), secondary hypertension, and hypothyroidism. He had four previous surgeries (living donor kidney transplant-2002, thyroidectomy and parathyroidectomy- in 2019, and two hemodialysis fistulas). His chronic treatment consisted of Levothyroxine sodium (Euthyrox, Merck) 100 μg/day, folic acid 5 mg/day, and micronized purified flavonoid fraction (Detralex, Les Laboratoires Servier) 1,000 mg/day.

He had a recent SARS-COV2 infection (with a positive RT-PCR from 2nd of September 2022), with minimal symptoms (low-grade fever present for 24 h and accentuated fatigability, compared to his normal state) treated with 5 days of Molnupiravir.

During 2020 and 2021, he was vaccinated three times with BioNTech and Pfizer vaccines and had a COVID-19 infection in 2021.

The perioperative chest auscultation revealed no pathological findings. The chest X-rays showed minimal changes: a slightly accentuated pulmonary interstitium and first-grade pulmonary hypertension changes. The preoperative arterial blood gas analysis showed normal oxygenation (PaO_2_ = 89.5 mm Hg) and normocapnia (PaCO_2_= 34.1 mm Hg) in room air. The preoperative cardiac evaluation showed a normal ECG, contractility pattern, average ejection fraction, and no valvopathies. The abdominal ultrasound showed bilateral atrophic kidneys, renal cysts, and a difficult-to-assess renal graft in the right flank.

The patient's preoperative bloodwork showed elevated BUN (22.83 mmol/L), creatinine (1027.44 μmol/L) and potassium (5.8 mmol/l), a mild increase in pancreatic amylase (249 U/l) and a low calcium level (3.78 mmol/l), average parathormone level (6.1 pg/ml), normal C-Reactive Protein (CRP) and slightly elevated Procalcitonin (PCT) level (0.19 ng/ml). Negative CDC cross matches four mismatches, calculated panel reactive antibody (cPRA) of 86%, no donor-specific antibody (DSA).

Following the evaluation performed by the transplant committee, he was informed of the potential risks associated with anesthesia and surgery after a recent COVID-19 infection; informed consent was obtained. Following the induction of anesthesia he was hemodynamically monitored throughout the surgery, using a non-invasive technique (ClearSight system^^®^^, Edwards Lifesciences): cardiac output (CO), stroke volume variation (SVV), pulse pressure variation (PPV) and systemic vascular resistances (SVR) were used in guiding fluid therapy, vasoactive and inotrope pharmacological support. Due to a low CO with normal SVV, PPV, and SVR following the induction, a low dose dopamine infusion (1.8–3.5 μg/kg/min) was initiated to maintain an adequate CO and aiming to ensure organ perfusion and avoid possible ischemic and thrombotic complications. Fluids were restricted, as all preload parameters were in the normal range and had 2.9 kg over de dialysis ideal body weight. The induction immunosuppressant therapy comprised Basiliximab (20 mg day 0 and day 4) and Methylprednisolone (1 g), as established by the hospital's transplant protocol. No surgical complications were noted during the surgery. Cold ischemia time was < 12 h and transplant duration 120 min.

The immediate postoperative period was marked by delayed graft function requiring one hemodialysis session on the 5th day after the surgery, with increasing urinary output (from 0.5 ml/kg/h immediately after transplantation to 3 ml/kg/h on the 3rd day), progressive decrease in creatinine level up to 194.5 μmol/L. Postoperatively he develops thrombocytopenia (ranging between 75 × 10^3^ and 120 × 10^3^) and leukopenia starting on the 6th day after transplantation.

Maintenance immunosuppressive therapy included Mycophenolic acid with adjusted doses due to leukopenia, tacrolimus with trough levels of 8 ng/ml and methylprednisolone.

Prophylactic treatment with trimethoprim-sulfamethoxazole and valganciclovir in renal doses was also undergone in the postoperative period.

Two weeks after transplantation, the patient complained of abdominal pain, loss of appetite, weakness and his bloodwork showed elevated inflammation markers (CRP = 2.43 mg/dl) and increased lipase and amylase levels (2,398 U/L, 2,776 U/L). The abdominal CT scan showed acute edematous pancreatitis with a CT severity index of 2, corresponding to mild pancreatitis. It also revealed a 2 mm calculus of the gallbladder. The laboratory findings showed elevated liver enzymes [ASAT, ALAT, gamma-glutamyl transferase (GGT)]. There were no pathological findings on the chest CT scan.

Oral alimentation was stopped and replaced by crystalloids and IV solutions with glucose, amino acids, and albumin, with clinical and biological improvement. After 5 days of fasting, oral feeding was gradually initiated with no rebound symptomatology. The patient was discharged on the 28th day after the kidney transplant surgery.

## Discussion

There have been several reports of successful kidney transplantation following COVID-19 infection, but in all cases, the patients tested negative at RT-PCR for the virus at the time of the surgery and had more than 4 weeks from the diagnosis of the viral infection (4–8). The particularity of our case consists in the fact that our patient, a hyper-immunized one, had only 2 weeks from the diagnosis of SARS CoV infection, with no indication for RT-PCR re-testing due to the period of fewer than 90 days from the onset of symptoms. From the anesthetic point of view, this patient was no longer considered infectious since he had more than 10 days from the beginning of the illness.

The best timing for surgery after COVID-19 infection is still controversial since most of the available data come from study periods with zero or low prevalence of vaccination and COVID-19 variants with high mortality rates. In addition, published data suggest that vaccinated patients experience lower risks of postoperative complications than unvaccinated patients (9). Nowadays, protocols are based on limited data specific to SARS-CoV-2, expert opinion, and previous data from other post-viral syndromes (10). The American Society of Anesthesiology recommends that elective surgery be delayed for 7 weeks after a SARS-CoV-2 infection in unvaccinated patients that are asymptomatic at the time of surgery and considers the existing data to be insufficient to make a recommendation for vaccinated patients [ASA]. Furthermore, ASA's statement underlines the necessity of weighing the risks and benefits of further delaying a surgery against the risks and benefits of having the surgery (10). In our case, we considered that the patient was a hyper-immunized, re-transplant one, with a low chance of graft allocation, against the risks of developing pulmonary and systemic complications due to a recent COVID-19 infection. The patient was informed of the risks and benefits, and a joint decision was made. Even though there were only 2 weeks from the mild COVID-19 diagnosis, the patient was completely asymptomatic, with minimal pathological findings on the chest X-ray, not suggestive of COVID involvement.

CDC guidelines no longer recommend re-testing for COVID-19 within 90 days of symptom onset. Repeated PCR testing in asymptomatic patients is discouraged since persistent positive PCR tests are expected after the illness.^1^ Moreover, antigen testing is advised in the recovery period after COVID-19 if a recurrence of symptoms is experienced (see text footnote 1). In our case, the patient tested negative on admission at the antigen testing and had no signs of recurrence during hospital stay.

In the perioperative setting, the amylase levels were twice the average (249 U/L). As our patient was completely asymptomatic, we interpreted this value as being in the context of chronic kidney disease and creatinine clearance below 50 ml/min (11). Two weeks after the kidney transplantation, the patient experienced AP clinical symptoms accompanied by increased serum pancreatic enzymes and characteristic changes on the CT scan. The Atlanta criteria were met, and several possible causes were considered. After excluding hepatitis B and C, cytomegalovirus, Epstein–Barr virus, alcohol, hypercalcemia, hypertriglyceridemia, hyperparathyroidism, and ischemia as possible causes of AP, we were left with the possibility of biliary, drug-induced and COVID 19 associated acute pancreatitis.

In our case, there was a prior diagnosis of a gallbladder stone (2 mm), with no history of gastrointestinal symptoms or increased pancreatic enzyme levels. This chronic pathological finding was not considered to be a contraindication for transplantation. Considering the high incidence of biliary pancreatitis and the presence of gallbladder stones, this diagnosis made the most probable cause of AP.

Drug-induced acute pancreatitis was also considered. Sulfa drugs, steroids, and proton pump inhibitor (PPI), potential triggers for AP, were part of the treatment plan. The Naranjo adverse drug reaction probability scale was 2, showing a possible drug reaction (12). The incriminated medication was not stopped during the episode of acute pancreatitis since there were no alternatives to pneumocystis carinii prophylaxis; the steroid dose was lowered.

The gastrointestinal tract may be affected during COVID-19 infection, in a range between 3 and 79% (13). The exocrine function of the pancreas was described to be altered in SARS-CoV-2 infection, some studies highlighting the presence of associated hyperenzymemia, while others describing isolated and rare cases of acute pancreatitis; all cases of acute pancreatitis were found in severe or critical forms of COVID 19, and none in mild and moderate conditions (14). Furthermore, in a recent article, the authors underlined that the seriousness of acute pancreatic inflammation increases with the degree of severity of lung involvement (14). In light of these findings, COVID-19-associated pancreatitis was considered an unlikely diagnosis.

Regarding the hyperimmune state of the patient and the ability of COVID-19 vaccination to induce anti-HLA antibody (Abs) formation in renal transplant candidates, we consider that more studies are required in order to better understand this phenomena. Some reports evidence that COVID-19 vaccination could be associated with anti-HLA Abs formation in renal patients on waitlists that could affect transplant eligibility (15).

Our patient received two doses of the mRNA vaccine. Booster vaccines enhance waning immunity and expand the breadth of immunity against SARS-CoV-2 variants of concern. Clinical trials have shown that receipt of a booster that does not match the primary vaccination (heterologous booster) may result in a higher neutralizing-antibody response than a matching (homologous) booster (16–18).

But, unvaccinated kidney transplant recipients who develop COVID-19 have a mortality of 20–40% (19) and SARS-CoV-2 vaccination is far less effective after kidney transplantation than during dialysis (20). To offer kidney transplantation without SARS-CoV-2 vaccination could be unsafe for the kidney recipient patient. The vaccine is indicated to the patients from the waiting list considering the risk-benefit analysis of the COVID-19 vaccination.

Our patient had a previous transplant as a source of immunization; this status was documented pre-pandemic with no modification of the panel anti-HLA antibodies after vaccination.

## Conclusion

Since cadaveric is not an elective surgery, the decision to undergo transplantation must be weighed against the patient's clinical needs. Our case suggests that we can safely proceed with kidney transplantation earlier than recommended in vaccinated, asymptomatic patients with COVID-19 variants with lower mortality rates. Further research is needed to support this statement.

## Data availability statement

The datasets presented in this article are not readily available. Requests to access the datasets should be directed to antal.oanna@gmail.com.

## Ethics statement

Ethical review and approval was not required for the study on human participants in accordance with the local legislation and institutional requirements. The patients/participants provided their written informed consent to participate in this study. Written informed consent was obtained from the participant/patient(s) for the publication of this case report.

## Author contributions

OA: anesthesia and article writing. AD: nephrologist and transplantation nephrologist. AM: nephrologist and transplantation expertise. TM and FE: transplantation surgeon and article revision. RM: tissue transplantation surgeon and article writing. All authors contributed to the article and approved the submitted version.

## Footnotes

^1^Available online at: https://www.cdc.gov/coronavirus/2019-ncov/hcp/testing-overview.html (accessed October 15, 2022).
